# Dental anomalies in craniofacial microsomia: A systematic review

**DOI:** 10.1111/ocr.12351

**Published:** 2019-10-28

**Authors:** Eline E. C. M. Elsten, Cornelia J. J. M. Caron, David J. Dunaway, Bonnie L. Padwa, Chris Forrest, Maarten J. Koudstaal

**Affiliations:** ^1^ Department of Oral and Maxillofacial Surgery The Dutch Craniofacial Centre Erasmus University Medical Center Sophia’s Children’s Hospital Rotterdam Rotterdam The Netherlands; ^2^ The Craniofacial Unit Great Ormond Street Hospital London UK; ^3^ The Craniofacial Centre Boston Children’s Hospital Boston MA USA; ^4^ The Center for Craniofacial Care and Research SickKids Hospital Toronto Ontario Canada

**Keywords:** craniofacial microsomia, dental anomalies, systematic review

## Abstract

Objective: To provide an overview on the prevalence and types of dental anomalies in patients with craniofacial microsomia (CFM). Eligibility criteria: Inclusion criteria were CFM and dental anomalies. The following data were extracted: number of patients, methodology, mean age, sex, affected side, severity of mandibular hypoplasia, dentition stage and dental anomalies. Information sources: Cochrane, EMBASE, PubMed, MEDLINE Ovid, Web of Science, CINAHL EBSCOhost and Google Scholar, searched until the 30 August 2019. Risk of bias: The quality was examined with the OCEBM Levels of Evidence. Included studies: In total, 13 papers were included: four retrospective cohort studies, four prospective cohort studies, four case‐control studies and one case series. Synthesis of results: The studies reported information on dental agenesis, delayed dental development, tooth size anomalies, tooth morphology and other dental anomalies. Description of the effect: Dental anomalies are more often diagnosed in patients with CFM than in healthy controls and occur more often on the affected than on the non‐affected side. Strengths and limitations of evidence: This is the first systematic review study on dental anomalies in CFM. However, most articles were of low quality. Interpretation: Dental anomalies are common in CFM, which might be linked to the development of CFM. The pathophysiology of CFM is not entirely clear, and further research is needed.

## 1 INTRODUCTION

In patients with craniofacial microsomia (CFM), the development of the first and second pharyngeal arches during the first 6 weeks of gestation is disturbed, resulting in diverse craniofacial malformations, including underdevelopment of the maxilla, mandible, ears, facial muscles and nerves.^1^, ^2^ CFM is the second most common craniofacial birth defect with an incidence varying between 1 in 3500 and 1 in 45 000^3^, ^4^, ^5^, ^6^ and is considered to be a unilateral condition. However, 10% of the patients with CFM are affected bilaterally.^7^, ^8^

The phenotype of CFM is heterogeneous as is demonstrated by the many terms used for describing CFM, for example first and second branchial arch syndrome, hemifacial microsomia, oculoauriculovertebral dysplasia and otomandibular dysostosis.^9^, ^10^, ^11^ Despite this heterogeneity, 89%‐100% of the patients with CFM present with mandibular hypoplasia, the most prevalent anomaly of CFM. To describe the severity of mandibular hypoplasia and other malformations in CFM, several classification systems have been developed, that is the OMENS classification including the Pruzansky‐Kaban score, the Chierici score and the SAT scale.^12^, ^13^, ^14^, ^15^, ^16^

The OMENS classification is used to describe the anomalies of the orbit, mandible, ear, facial nerve and the soft tissues, whereby the Pruzansky‐Kaban score is used to determine the severity of the mandibular hypoplasia.^16^ Another classification system is the SAT scale and is used to describe the mandibular, auricular and soft‐tissue deformities that might occur in CFM.^14^ Moreover, mandibular deformities in CFM are as well described by the Chierici score.^15^ Using classification models, patients can be systematically described and categorized to provide insight into severity of the deformities and possible need for (surgical) treatment.

As a result of disturbed development of the first pharyngeal arch and maxillomandibular hypoplasia, dental anomalies might be expected in patients with CFM. A few studies describe tooth agenesis, hypodontia and delayed tooth development; however, not much literature is available on type and prevalence of dental anomalies in patients with CFM.^17^, ^18^, ^19^, ^20^

Dental anomalies can have various consequences. For example, dental agenesis can cause problems such as less alveolar bone growth, less functioning masticatory muscles and delayed dental development of the permanent dentition when primary teeth are agenetic.^21^, ^22^, ^23^, ^24^ Furthermore, delayed dental development can interfere with the planned orthodontic and orthognathic treatment.^25^ Also, absent or malformed teeth play an important role in the self‐esteem of patients and are a primary reason to choose treatment.^21^

Thus far, no systematic review is conducted regarding dental anomalies in CFM. To gain more insight on dental anomalies in CFM, a systematic review was conducted. The aim of this systematic review is to provide an overview of the literature regarding CFM and the prevalence and types of dental anomalies.

## 2 METHODS

### 2.1 Data sources and search strategy

The Preferred Reporting Items for Systematic Reviews and Meta‐Analyses (PRISMA) statement was used to guide this study.^26^ This study was accepted by the Erasmus Medical Center Medical Ethics Committee in 2013 and was not registered on any online website or database. A search of public domain databases was performed to identify all papers regarding CFM and its synonyms combined with dental anomalies and its synonyms. The search was conducted in the following databases: Cochrane, EMBASE, PubMed, MEDLINE Ovid, Web of Science, CINAHL EBSCOhost and Google Scholar, all searched up to 30 August 2019. In addition, a manual search of secondary sources including references of the papers was performed. Results were limited to human studies, implemented in the initial search. The full search terms are to be found in the Appendix 1 of this review.

### 2.2 Eligibility criteria

If patients had CFM and any form of dental anomalies, they were included in this research. Patients with isolated microtia were not considered as CFM and excluded from this study. The primary search (eg, title and abstract) had as limitation only human studies. The secondary search (eg, full text) had as limitation only human studies and articles more recent than 1980. The articles had to be available in English, German or Dutch full text or with English, German or Dutch summary or tables.

### 2.3 Data extraction and analysis

Two reviewers (EECME and CJJMC) independently screened the studies.

First, papers were included or excluded based on title and abstract. Inclusion criteria were (a) CFM and (b) dental anomalies. All papers on type, prevalence and/or treatment of dental anomalies in CFM were included and reviewed by full text. Papers for which the title and/or abstract was lacking information were reviewed by full text as well.

Next, inclusion or exclusion of papers based on full text was performed. Inclusion criteria were (a) CFM and (b) dental anomalies. Exclusion criteria were as follows: (a) expert opinions; (b) meeting abstracts, oral and/or poster presentations; (c) letters to the editor; (d) non‐English, non‐German or non‐Dutch papers without English/German/Dutch full text, summary or tables; (e) published before 1980; (g) incomplete data; and (h) case reports and/or series with up to three patients.

From every study included in this review, the following study characteristics were extracted and tabulated when available: number of patients, methodology, mean age of the patients, sex, affected side, severity of mandibular hypoplasia, dentition stage and the types and prevalence of dental anomalies. The types of dental anomalies investigated in this study were dental agenesis, delayed dental development, tooth size anomalies, tooth morphology anomalies and “other” dental anomalies, that is impacted teeth, interdental spacing, neonatal teeth and supernumerary teeth.

All studies were graded on quality by using the Oxford Centre for Evidence‐Based Medicine (CEBM) criteria.^27^

### 2.4 Mandibular hypoplasia

To describe the severity of mandibular hypoplasia in a uniform way, the terms mild, moderate and severe were conducted and used in this systematic review. Mild mandibular hypoplasia was used to describe mandibles with a Pruzansky‐Kaban I classification, a Chierici I score or a score I on the SAT scale. A Pruzansky‐Kaban classification of II, that is IIa and IIb, a Chierici score II or III, or a score II on the SAT scale were considered as moderate mandibular hypoplasia, and a Pruzansky‐Kaban III classification, Chierici IV or V score and a score III on the SAT scale were considered as severe mandibular hypoplasia.

### 2.5 Dental age

To describe the dental development stage of teeth, the articles included in this review used several measuring instruments: Nolla's stages of tooth calcification, dental development by Demirjian and tooth maturation by Demirjian and Goldstein.^28^, ^29^, ^30^ Nolla's stages of tooth calcification describe 10 stages of tooth development: 0 meaning absence of crypts, 10 meaning apical end of root completed. Everything in between is a stage of development of the tooth. The Demirjian Dental Development rates 7 teeth by a developed procedure. The stages were rated 0 for no calcification and A to H for the eight calcification stages. The score will be converted in a table. The scores for all seven teeth measured will give the maturity score, which can be plotted in centile charts. The Demirjian and Goldstein new system for dental maturity is based on the Demirjian Dental Development assessment, but scored with four teeth instead of seven.

## 3 RESULTS

### 3.1 Study selection

**Figure 1:**
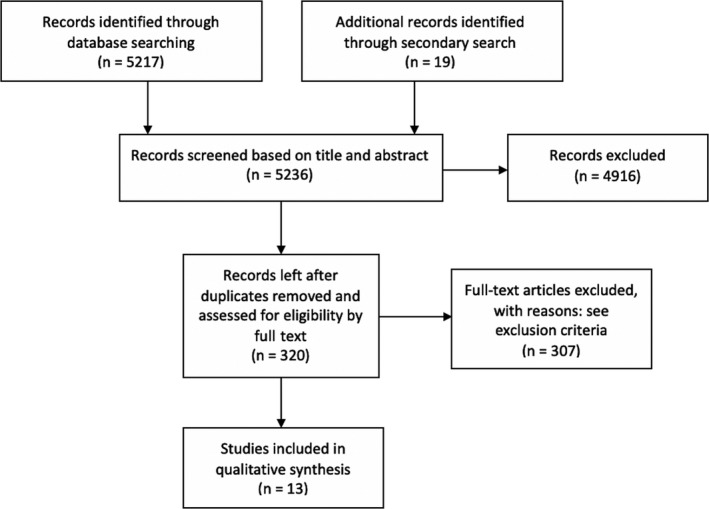
Data extraction flowchart

In total, 5236 papers were identified of which 4916 did not meet the inclusion criteria. The remaining 320 papers were reviewed by full text. In total, after further exclusion, 13 papers were included in this review for further analysis (Figure 1). The list of screened papers is available on request.

### 3.2 Study characteristics

In Tables 1 and 2, the study characteristics of the studies included in this review are described. The total number of CFM patients included in the studies ranged from 4 to 125. The age range of the patients varies from 0 to 43 years; however, not all studies reported the age of the patients. In three studies, the severity of the mandibular hypoplasia was not described. Overall, five studies included only unilaterally affected patients, three studies included both unilaterally and bilaterally affected patients, and in five studies, the laterality was not mentioned. In five studies, more males than females were included, in five studies, more females than males were included, and in two studies, both males and females were equally included. One study did not report gender. Four studies reported the dentition stage of the patients; most patients were in their primary or mixed dentition phase. The examinations used to determine dental anomalies were clinical examinations, facial photographs, dental casts, panoramic X‐rays, cephalograms, computed tomography scans and 3D scans.

**Table 1:** Study characteristics

Study	OCEBM levels of evidence	Methodology	Aim	Discussed dental anomalies
Ahiko et al^34^	III	Retrospective cohort study	To characterize maxillofacial morphology and dental development in patients with unilateral CFM	Dental agenesis Dental development
Chang et al^37^	III	Retrospective cohort study	To investigate the differences of primary and permanent teeth dimensions in the maxillary and mandibular dentition between the affected and non‐affected side in CFM patients	Tooth size
Farias et al^18^	III	Retrospective cohort study	To investigate the development of the dentition in patients with varying degrees of CFM	Dental agenesis Dental development
Farias et al^38^	III	Retrospective cohort study	To determine whether tooth size and morphology are affected in CFM	Tooth size Tooth morphology
Jacobsson et al^32^	II	Prospective cohort study	To investigate the clinical appearance of patients with mandibulofacial dysostosis, CFM and thalidomide‐induced malformations	Dental agenesis Other dental anomalies
Johnsen et al^39^	IV	Case series	To report enamel defects in 4 children with CFM	Tooth morphology
Kim Seow et al^36^	III	Case‐control study	To examine the primary and permanent tooth dimensions of dental casts of CFM patients	Tooth size
Loevy et al^33^	IV	Case‐control study	To evaluate dental development and maturation in CFM	Dental agenesis Dental development
Maruko et al^19^	IV	Case‐control study	To describe the patterns and prevalence of missing teeth in patients with CFM	Dental agenesis
Ongkosuwito et al^17^	IV	Case‐control study	To compare dental development scores between the affected and non‐affected side in CFM	Dental agenesis Dental development
Silvestri et al^31^	III	Prospective cohort study	To evaluate the incidence of agenesis and impacted teeth in CFM patients	Dental agenesis Other dental anomalies
Takashima et al^35^	III	Prospective cohort study	To test several hypotheses in CFM regarding the masticatory muscles	Dental agenesis Dental development
Touliatou et al^40^	III	Prospective cohort study	To present clinical manifestations in 17 patients with clinical diagnosis of CFM	Other

**Table 2:** Study characteristics

Source	No. of patients	Mean age (age range) in years	Sex (M/F)	Affected side (R/L/B)	Severity of mandibular hypoplasia (no. of patients)	Dentition stage (no. of patients)	Used dental examinations
Ahiko et al^34^	24	9.3 (4.3‐20.6)	12/12	15/9/0	Mild 17 Moderate 5 Severe 2	Primary 0 Mixed 24 Permanent 0	Cephalogram Panoramic X‐ray
Chang et al^37^	34	5.11	18/16	14/20/0	Mild 24 Moderate 7 Severe 3	Primary 23 Mixed 8 Permanent 3	NR
Farias et al^18^	60	(6‐24)	24/36	NR	Mild 26 Moderate 7 Severe 27	NR	Panoramic X‐ray
Farias et al^38^	40	(8‐21)	NR	NR	Mild 18 Moderate 4 Severe 18	NR	Dental casts Radiographic examination n.o.s.
Jacobsson et al^32^	26	26.3	12/14	16/10/0	NR	NR	Clinical examination CT scan
Johnsen et al^39^	4	NR	3/1	2/1/1	NR	NR	Clinical examination
Kim Seow et al^36^	50	NR	25/25	27/23/0	Mild 15 Moderate 18 Severe 13 Unknown 4	Primary dentition 20 Mixed dentition 15 Permanent dentition 15	Dental casts
Loevy et al^33^	89	7.8 (3.3‐13)	58/31	NR	Mild 57 Moderate 26 Severe 6	Primary dentition 0 Mixed dentition 89 Permanent dentition 0	Cephalogram Panoramic X‐ray
Maruko et al^19^	125	10.9 (4‐32)	65/60	56/59/3	Mild 52 Moderate 59 Severe 14	NR	Panoramic X‐ray
Ongkosuwito et al^17^	84	10.0 (3.1‐31)	37/47	NR	Mild 23 Moderate 53 Severe 8	NR	Panoramic X‐ray
Silvestri et al^31^	63	18.7 (7‐43)	27/36	36/25/2	Mild 21 Moderate 31 Severe 11	NR	Clinical examination Cephalogram CT scan
Takashima et al^35^	10	10.3 (6.9‐14.8)	4/6	5/5/0	Mild 4 Moderate Severe 6	NR	Facial photographs Dental casts Cephalogram Panoramic X‐ray CT scan 3D scan
Touliatou et al^40^	17	(0‐23)	10/7	NR	NR	NR	Clinical examination

Abbreviations: B = bilateral; CT = computed tomography scan; F = female; L = left; M = male; n.o.s., not otherwise specified; No., number; NR = not reported; R = right.

### 3.3 The prevalence and types of dental anomalies in CFM

#### 3.3.1 Dental anomalies overall

None of the 13 studies included in this review described a complete spectrum of dental anomalies in CFM. Therefore, no prevalence of dental anomalies overall in CFM could be given. Eight studies investigated dental agenesis in CFM. Dental development and tooth size were described in five and three studies, respectively. Tooth morphology anomalies were described in two studies, and neonatal teeth, impacted teeth, spacing and enamel defects were described in one study.

#### 3.3.2 Dental agenesis

In Table 3, the studies reporting dental agenesis are presented. The prevalence of dental agenesis varied from 6.7% to 33.3%. Maruko et al compared their results with a control population. The control population consisted of 45 subjects selected from an orthodontic clinic and was compared to the population of CFM patients who met the same criteria (ie, age 8 or older and at least one panoramic radiograph). Maruko et al found significant more dental agenesis in patients with CFM, with a prevalence of 26.9% in patients with CFM and no dental agenesis in the control population.

**Table 3:** Dental agenesis

Author	No. of patients	No. of affected patients (%)	Third molars included	1st most likely to be agenetic (no. of teeth)	2nd most likely to be agenetic (no. of teeth)
Ahiko et al^34^	24	8 (33.3)	No	LI2 (n = 6)	UPM2 (n = 3)
Farias et al^18^	60	15 (25)	Yes	LM3 (n = 26)	LPM2 (n = 9)
Jacobsson^32^	26	8 (30.8)	Yes	PM2 (n = 5)	PM1 (n = 4)
Loevy et al^33^	89	6 (6.7)	No	LPM2 (n = 9)	None
Maruko et al^19^	76^a^	25 (32.9)	No	LPM2 (n = 11)	UM2 and LM2 (both n = 7)
Ongkosuwito et al^17^	84	10 (32.9)	NR	NR	NR
Silvestri et al^31^	63	11 (17.4)	Yes	UM3 and LM3 (both n = 8)	UPM2 (n = 4)
Takashima et al^40^	10	NR	NR	NR	NR

Abbreviations: NR, not reported; U, upper (maxillary); L, lower (mandibular); I, incisor; PM, premolar; M, molar; 1, first; 2, second; 3, third.

a Seventy‐six out of the total of 125 included patients were 4 y of age or older and had a panoramic radiograph available; thus, only 76 patients were included in this analysis.

Mandibular third molars were most frequently missing, in 55.1%‐55.2% of the total amount of teeth being agenetic.^18^, ^31^ When the third molars were excluded from the analyses, the maxillary and/or mandibular second premolar were most frequently agenetic, varying from 11.0% to 100% of the total number of dental agenesis.^18^, ^19^, ^31^, ^32^, ^33^ In the studied cohort by Ahiko et al, the most frequently missing tooth was the mandibular lateral incisor. Loevy et al solely studied agenesis in the mandible, and Jacobsson et al did not differentiate between the mandible and maxilla.

In five studies, the difference in dental agenesis between the affected and non‐affected side was described. According to one study, dental agenesis was significantly more often diagnosed on the affected side than on the unaffected side.^18^ Three studies reported more dental agenesis on the affected side as well; however, the statistics and significance were not reported in these studies.^19^, ^31^, ^33^ Additionally, dental agenesis was also more frequently diagnosed in patients with bilateral CFM than in patients with unilateral CFM.^31^, ^33^

Dental agenesis was more frequently diagnosed in patients with more severe mandibular hypoplasia than in patients with less severe mandibular hypoplasia.^17^, ^19^, ^31^, ^33^ However, in the studies by Ongkosuwito et al and Maruko et al, these results were significant. In the other studies, the significance was not mentioned. As a result, there is no statement on the significance of that result.

#### 3.3.3 Delayed dental development

The characteristics of the studies describing delayed dental development are shown in Table 4. The reported prevalence of delayed dental development in CFM varied from 20.5% to 54.3%. To measure delayed dental development, Nolla's stages of tooth calcification, the Demirjian Dental Age Assessment and the tooth maturation by Demirjian and Goldstein were used.^28^, ^29^, ^30^

**Table 4:** Dental development

Author	Measuring instrument	No. of patients	No. of affected patients (%)
Ahiko et al^34^	Nolla's stages of tooth calcification (Nolla 1960)	24	5 (20.8)
Farias et al^18^	Nolla's stages of tooth calcification	60	30 (50)
Loevy et al^33^	Tooth maturation by Demirjian and Goldstein (1976)	81	44 (54.3)
Ongkosuwito et al^17^	Dental development by Demirjian (1973)	84	34 (40.4)
Takashima et al^35^	Dental development by Demirjian (1973)	10	NR

Delayed dental development was found to occur significantly more frequently on the affected side than on the non‐affected side in two studies.^18^, ^34^ Three studies did not find a significant result comparing the affected and non‐affected side regarding dental development.^17^, ^33^, ^35^ Additionally, in patients with CFM, delayed dental development was significantly more often diagnosed than in the control population, that is healthy children without (craniofacial) syndromes.^17^, ^33^

Of the total number of teeth being affected, the mandibular second and third molars were most frequently affected according to Farias et al and Ahiko et al, with 43.9% and 36.6%, respectively, reported by Farias et al.^18^, ^34^

Interestingly, Ongkosuwito et al found a “catch‐up phenomenon” in patients with delayed dental development. Although earlier in life delayed dental development occurred more often in CFM patients compared with controls, later in life the development is faster than the norm, suggesting that there is a reduction in developmental delay.^17^

#### 3.3.4 Tooth size anomalies

Three studies investigated the mesiodistal width of both the maxillary and mandibular teeth. Kim Seow et al described the points of contact with the adjacent teeth to measure the mesiodistal width.^36^ Chang et al and Farias et al mentioned measuring the mesiodistal crown width; however, the exact used points were not further specified.^37^, ^38^ All studies found that the permanent mandibular first molar was significantly smaller on the affected side than the mandibular first molar on the unaffected side. However, according to Farias et al, this result was only significant in patients with more severe mandibular hypoplasia.^36^, ^37^, ^38^

Additionally, primary mandibular second molars and permanent mandibular canines were also found to be smaller on the affected side than on the unaffected side. Moreover, Kim Seow et al described significantly smaller primary maxillary first and second molars, primary mandibular first molars and permanent maxillary first molars when the affected and non‐affected side were compared.^36^ As for the other teeth, a significant difference in tooth size between the affected and non‐affected side was not found.

Interestingly, Kim Seow et al compared the results of mesiodistal width in patients with CFM, with a control population, that is healthy children without (craniofacial) syndromes, and found that the primary maxillary and mandibular first and second molars, the permanent maxillary and mandibular first molars were significantly smaller in patients with CFM than in the control population.^36^

The faciolingual width of teeth was investigated by Chang et al and Kim Seow et al did this by measuring the distance between the centre of the buccal and palatinal/lingual gingival margins.^36^, ^37^ In the study by Chang et al, the exact used method remained unknown. The faciolingual width did not show any significant differences between patients with and without CFM and between the affected and unaffected side.^37^

#### 3.3.5 Tooth morphology

Two studies found anomalies in tooth morphology in patients with CFM. Farias et al^38^ reported that 6 out of 40 (15%) patients had four‐cusp first molars on the affected side and five‐cusp first molars on the non‐affected side. This was found in patients who had a more severe mandibular hypoplasia. Enamel hypoplasia and opacities were clinically investigated in patients and reported by Johnsen et al.^39^ In the primary dentition of four patients, these enamel defects were found, more often on the affected side than on the non‐affected side.

#### 3.3.6 Other

Jacobsson et al^32^ reported multiple neonatal teeth in one patient. Tooth impaction was noted by Silvestri et al in 5 out of 63 patients (7.9%), all but one on the affected side. However, this was mainly found in patients with less severe mandibular hypoplasia and significance was not described.^31^ Touliatou et al^40^ reported excessive interdental spacing in 2 out of 17 patients (11.8%). In one of the cases with enamel defects, described by Johnsen et al^39^, a supernumerary left maxillary lateral incisor was also found.

## 4 DISCUSSION

### 4.1 The prevalence and types of dental anomalies in CFM

The aim of this systematic review was to describe the types and prevalence of dental anomalies in CFM. The reported prevalence of dental agenesis in patients with CFM varied from 6.7% to 33.3%; these prevalence rates are higher than those reported in the general population, which varies from 4.5% to 13.3%.^41^, ^42^, ^43^, ^44^, ^45^, ^46^, ^47^, ^48^, ^49^, ^50^ When dental agenesis occurs in patients with CFM and when third molars are excluded from analyses, the most likely teeth to be missing are the second premolars. The same result is found in healthy controls with dental agenesis.^41^, ^42^, ^44^, ^46^, ^47^, ^48^, ^49^, ^50^

With a prevalence varying between 20.0% and 54.4%, delayed dental development was found more often in patients with CFM than in the healthy population, in which delayed dental development occurs in 3.4%‐4.3%.^25^, ^51^ Furthermore, delayed dental development occurred more frequently on the affected side than on the unaffected side.

Although the prevalence of smaller tooth size in patients with CFM was not reported, CFM patients have smaller molars compared to healthy controls. In addition, CFM patients have smaller permanent and primary mandibular and maxillary first molars and when the affected and non‐affected side were compared. Moreover, other findings in this review regarding dental anomalies in CFM were four‐cusp first molars, enamel defects, neonatal teeth, tooth impaction and interdental spacing.

As mentioned above, dental anomalies are common in CFM and are more often found in patients with CFM than in the healthy population. These dental anomalies might be caused by a disturbance in the development of the first pharyngeal arch, as CFM is the result of a disturbance of the first (and second) pharyngeal arch as well.^1^, ^2^ Dental tissue derives from the first pharyngeal arch and starts to develop in the 6th week of gestation.^52^, ^53^ As a result of a disturbance in the development of the first and second pharyngeal arch in patients with CFM, incomplete or abnormal dentition, or delayed development of dentition can be the consequence.^54^

The cause of this disturbance in the development of the face and dentition in CFM is still unknown. A theory is that stapedial artery damage in embryos causes a haematoma that disturbs the normal development of the branchial arches.^55^, ^56^ Environmental factors such as maternal diabetes, hypoxia and teratogens such as thalidomide might also play part.^57^, ^58^, ^59^, ^60^, ^61^

Moreover, not only environmental factors but also genetic factors could cause CFM and its dental anomalies. One of the theories of the origin of CFM is that defects during the neural crest cell migration might cause the craniofacial anomalies seen in CFM.^62^, ^63^ Since neural crest cells are also involved in dental development, the defect in neural crest cell migration might also lead to dental anomalies.^64^ Furthermore, for example a disturbed FGF pathway is in some non‐syndromic patients responsible for dental agenesis and disturbed tooth development.^65^ Literature suggests that FGF8 plays part in the development of the first branchial arch, and when the FGF pathway is disturbed, this might be involved in the underdevelopment of one side of the face.^66^ Last, two families with autosomal dominant CFM have been found with an affected 14q32 locus.^67^ Although dental anomalies were not described in these families, dental anomalies are described in non‐CFM patients with an affected 14q32 chromosome as well.^68^, ^69^

The presence of dental anomalies in CFM could also be a result of the more common presence of a cleft lip and palate (CLP). The studies included in this review did not report the presence of CLP. CLP can occur in CFM patients and can have a relation to dental anomalies as well.^70^, ^71^, ^72^, ^73^, ^74^, ^75^, ^76^, ^77^, ^78^ With this review, it is not possible to answer the question if dental anomalies are more likely to occur in CFM patients with CLP than in CFM patients without CLP.

Furthermore, previous studies suggest that there might be a connection between facial asymmetry and dental anomalies.^79^, ^80^, ^81^, ^82^ This review presents that dental anomalies are more common on the affected side than on the non‐affected side, although more often in more severely affected patients. However, the exact connection between facial unilateral hypoplasia and dental anomalies remains unclear with this review.

Also, the complete spectrum of dental anomalies, such as supernumerary teeth, dental exfoliation anomalies and dental resorption, is not described in the studies included in this review. The possibility exists that when more dental anomalies are investigated, this might affect the results.

To answer the questions regarding dental anomalies in CFM, further research is needed. Larger prospective and retrospective studies can be helpful to identify the problems that may occur in CFM and to provide better care for these patients. An international research collaboration is founded between the Erasmus Medical Center Rotterdam, the Great Ormond Street Hospital London, SickKids Hospital Toronto and Boston Children's Hospital to obtain more information about the problems that may occur in CFM.

### 4.2 Limitations of the study

This study has several limitations. First, some papers that were selected for assessment based on full text were not available in English, German or Dutch and did not have English, German of Dutch summaries and/or tables. It was not possible for the authors to read these papers and collect data from these papers. For this reason, there is a possibility that some data are missed in this review.

Second, the quality of the studies was medium level. Most studies were rated level III quality, and one study (Johnsen et al) was rated level IV because of the small patient group included. Before study selection, the authors decided to exclude studies with three patients or less from our review. Still, four patients is a small sample for a trustworthy analysis. The study by Takashima et al only mentioned that five patients had abnormal dentition, for example congenitally absent teeth, retention of permanent teeth or delayed maturation. This was not further specified, and therefore, no hard conclusions could be drawn from this study.

## 5 CONCLUSION

A systematic review was conducted to provide an overview regarding dental anomalies in CFM. Dental anomalies such as dental agenesis, delayed dental development and smaller tooth size are more common in patients with CFM than in healthy controls and occur more often on the affected side than on the non‐affected side. However, precise numbers and statistics are not given in most studies. Therefore, hard conclusions cannot be drawn from this study and further research is needed.

## CONFLICT OF INTEREST

All authors made substantial contributions to conception and design, acquisition of data or analysis and interpretation of data. All authors were involved in drafting the paper or critically revising it for important intellectual content. And, finally, authors approved the version to be published.

## AUTHORS CONTRIBUTIONS

All authors made substantial contributions to conception and design, acquisition of data, or analysis and interpretation of data. All authors were involved in drafting the paper or critically revising it for important intellectual content. And, finally, all authors approved of the version to be published.

## PATIENT CONSENT

Informed consent has been given in the original study.

## Appendix 1

### FULL SEARCH TERMS PER DATABASE

#### Embase.com

('face asymmetry'/exp OR 'Goldenhar syndrome'/de OR 'hemifacial microsomia'/de OR 'mandibulofacial dysostosis'/de OR (((facial OR face OR hemifacial OR orbitocranial OR facies OR cranial OR mandibulofacial OR otomandibular OR craniofacial OR faciocranial OR hemimandibular) NEAR/3 (microsom* OR asymmetr* OR dysosto* OR dysplasia OR anomal* OR deformit* OR hypoplasia OR syndrom* OR malformation*)) OR 'treacher collins' OR goldenhar OR oculoauriculovertebral* OR facioauriculovertebral* OR (auriculo NEXT/1 vertebral*)):ab,ti) AND ('tooth malformation'/exp OR 'tooth development'/exp OR 'tooth disease'/de OR (((tooth OR teeth OR dental OR enamel OR dentin* OR odont* OR molar* OR premolar* OR Incisor* OR canine OR crown OR cuspid* OR bicuspid* OR denture*) NEAR/6 (malform* OR abnormal* OR anomal* OR hypoplasia OR fused OR fusion OR supernumerar* OR deformit* OR defect* OR deficien* OR missing OR malposition* OR misplace* OR pulpa OR develop* OR asymmetr* OR eruption* OR formation* OR delay* OR unusual* OR impact* OR single OR solitar* OR sequence* OR crowd* OR aberration* OR aspect* OR manifestation* OR imperfect* OR absen* OR spacing OR alteration* OR size OR displace* OR characteristic* OR involve* OR maturat* OR incomplete* OR agenesis OR hypomineral* OR mineral*)) OR (root NEAR/3 resorption*) OR hypodonti* OR oligodonti* OR adonti* OR odontom* OR edentulous* OR dentition* OR dysplasi* OR odontodysplasi* OR macrodont* OR microdont* OR anodont* OR odontogen* OR amelogen*):ab,ti) NOT ([animals]/lim NOT [humans]/lim)

#### MEDLINE Ovid

(exp "Mandibulofacial Dysostosis"/ OR "Facial Asymmetry"/ OR (((facial OR face OR hemifacial OR orbitocranial OR facies OR cranial OR mandibulofacial OR otomandibular OR craniofacial OR faciocranial OR hemimandibular) ADJ3 (microsom* OR asymmetr* OR dysosto* OR dysplasia OR anomal* OR deformit* OR hypoplasia OR syndrom* OR malformation*)) OR "treacher collins" OR goldenhar OR oculoauriculovertebral* OR facioauriculovertebral* OR (auriculo ADJ vertebral*)).ab,ti.) AND (exp "Tooth Abnormalities"/ OR exp "Odontogenesis"/ OR "tooth disease"/ OR exp tooth/ab OR exp tooth/gd OR (((tooth OR teeth OR dental OR enamel OR dentin* OR odont* OR molar* OR premolar* OR Incisor* OR canine OR crown OR cuspid* OR bicuspid* OR denture*) ADJ6 (malform* OR abnormal* OR anomal* OR hypoplasia OR fused OR fusion OR supernumerar* OR deformit* OR defect* OR deficien* OR missing OR malposition* OR misplace* OR pulpa OR develop* OR asymmetr* OR eruption* OR formation* OR delay* OR unusual* OR impact* OR single OR solitar* OR sequence* OR crowd* OR aberration* OR aspect* OR manifestation* OR imperfect* OR absen* OR spacing OR alteration* OR size OR displace* OR characteristic* OR involve* OR maturat* OR incomplete* OR agenesis OR hypomineral* OR mineral*)) OR (root ADJ3 resorption*) OR hypodonti* OR oligodonti* OR adonti* OR odontom* OR edentulous* OR dentition* OR dysplasi* OR odontodysplasi* OR macrodont* OR microdont* OR anodont* OR odontogen* OR amelogen*).ab,ti.) NOT (exp animals/ NOT humans/)

#### Cinahl EBSCOhost

(MH "Mandibulofacial Dysostosis+" OR TI(((facial OR face OR hemifacial OR orbitocranial OR facies OR cranial OR mandibulofacial OR otomandibular OR craniofacial OR faciocranial OR hemimandibular) N2 (microsom* OR asymmetr* OR dysosto* OR dysplasia OR anomal* OR deformit* OR hypoplasia OR syndrom* OR malformation*)) OR "treacher collins" OR goldenhar OR oculoauriculovertebral* OR facioauriculovertebral* OR (auriculo N1 vertebral*)) OR AB (((facial OR face OR hemifacial OR orbitocranial OR facies OR cranial OR mandibulofacial OR otomandibular OR craniofacial OR faciocranial OR hemimandibular) N2 (microsom* OR asymmetr* OR dysosto* OR dysplasia OR anomal* OR deformit* OR hypoplasia OR syndrom* OR malformation*)) OR "treacher collins" OR goldenhar OR oculoauriculovertebral* OR facioauriculovertebral* OR (auriculo N1 vertebral*))) AND (MH "Tooth Abnormalities+" OR MH "tooth diseases" OR TI (((tooth OR teeth OR dental OR enamel OR dentin* OR odont* OR molar* OR premolar* OR Incisor* OR canine OR crown OR cuspid* OR bicuspid* OR denture*) N5 (malform* OR abnormal* OR anomal* OR hypoplasia OR fused OR fusion OR supernumerar* OR deformit* OR defect* OR deficien* OR missing OR malposition* OR misplace* OR pulpa OR develop* OR asymmetr* OR eruption* OR formation* OR delay* OR unusual* OR impact* OR single OR solitar* OR sequence* OR crowd* OR aberration* OR aspect* OR manifestation* OR imperfect* OR absen* OR spacing OR alteration* OR size OR displace* OR characteristic* OR involve* OR maturat* OR incomplete* OR agenesis OR hypomineral* OR mineral*)) OR (root N2 resorption*) OR hypodonti* OR oligodonti* OR adonti* OR odontom* OR edentulous* OR dentition* OR dysplasi* OR odontodysplasi* OR macrodont* OR microdont* OR anodont* OR odontogen* OR amelogen*) OR AB (((tooth OR teeth OR dental OR enamel OR dentin* OR odont* OR molar* OR premolar* OR Incisor* OR canine OR crown OR cuspid* OR bicuspid* OR denture*) N5 (malform* OR abnormal* OR anomal* OR hypoplasia OR fused OR fusion OR supernumerar* OR deformit* OR defect* OR deficien* OR missing OR malposition* OR misplace* OR pulpa OR develop* OR asymmetr* OR eruption* OR formation* OR delay* OR unusual* OR impact* OR single OR solitar* OR sequence* OR crowd* OR aberration* OR aspect* OR manifestation* OR imperfect* OR absen* OR spacing OR alteration* OR size OR displace* OR characteristic* OR involve* OR maturat* OR incomplete* OR agenesis OR hypomineral* OR mineral*)) OR (root N2 resorption*) OR hypodonti* OR oligodonti* OR adonti* OR odontom* OR edentulous* OR dentition* OR dysplasi* OR odontodysplasi* OR macrodont* OR microdont* OR anodont* OR odontogen* OR amelogen*)) NOT (MH animals + NOT MH humans+)

#### Cochrane

((((facial OR face OR hemifacial OR orbitocranial OR facies OR cranial OR mandibulofacial OR otomandibular OR craniofacial OR faciocranial OR hemimandibular) NEAR/3 (microsom* OR asymmetr* OR dysosto* OR dysplasia OR anomal* OR deformit* OR hypoplasia OR syndrom* OR malformation*)) OR 'treacher collins' OR goldenhar OR oculoauriculovertebral* OR facioauriculovertebral* OR (auriculo NEXT/1 vertebral*)):ab,ti) AND ((((tooth OR teeth OR dental OR enamel OR dentin* OR odont* OR molar* OR premolar* OR Incisor* OR canine OR crown OR cuspid* OR bicuspid* OR denture*) NEAR/6 (malform* OR abnormal* OR anomal* OR hypoplasia OR fused OR fusion OR supernumerar* OR deformit* OR defect* OR deficien* OR missing OR malposition* OR misplace* OR pulpa OR develop* OR asymmetr* OR eruption* OR formation* OR delay* OR unusual* OR impact* OR single OR solitar* OR sequence* OR crowd* OR aberration* OR aspect* OR manifestation* OR imperfect* OR absen* OR spacing OR alteration* OR size OR displace* OR characteristic* OR involve* OR maturat* OR incomplete* OR agenesis OR hypomineral* OR mineral*)) OR (root NEAR/3 resorption*) OR hypodonti* OR oligodonti* OR adonti* OR odontom* OR edentulous* OR dentition* OR dysplasi* OR odontodysplasi* OR macrodont* OR microdont* OR anodont* OR odontogen* OR amelogen*):ab,ti)

#### Web of science

TS=(((((facial OR face OR hemifacial OR orbitocranial OR facies OR cranial OR mandibulofacial OR otomandibular OR craniofacial OR faciocranial OR hemimandibular) NEAR/2 (microsom* OR asymmetr* OR dysosto* OR dysplasia OR anomal* OR deformit* OR hypoplasia OR syndrom* OR malformation*)) OR "treacher collins" OR goldenhar OR oculoauriculovertebral* OR facioauriculovertebral* OR (auriculo NEAR/1 vertebral*))) AND ((((tooth OR teeth OR dental OR enamel OR dentin* OR odont* OR molar* OR premolar* OR Incisor* OR canine OR crown OR cuspid* OR bicuspid* OR denture*) NEAR/5 (malform* OR abnormal* OR anomal* OR hypoplasia OR fused OR fusion OR supernumerar* OR deformit* OR defect* OR deficien* OR missing OR malposition* OR misplace* OR pulpa OR develop* OR asymmetr* OR eruption* OR formation* OR delay* OR unusual* OR impact* OR single OR solitar* OR sequence* OR crowd* OR aberration* OR aspect* OR manifestation* OR imperfect* OR absen* OR spacing OR alteration* OR size OR displace* OR characteristic* OR involve* OR maturat* OR incomplete* OR agenesis OR hypomineral* OR mineral*)) OR (root NEAR/2 resorption*) OR hypodonti* OR oligodonti* OR adonti* OR odontom* OR edentulous* OR dentition* OR dysplasi* OR odontodysplasi* OR macrodont* OR microdont* OR anodont* OR odontogen* OR amelogen*)) NOT ((animal* OR rat OR rats OR mouse OR mice OR murine OR sheep OR ovine OR cow OR bovine OR dog OR canine OR cat OR feline OR horse OR equine OR monkey OR primate* OR chimpan* OR gorilla*) NOT (human* OR patient*)))

#### Google scholar

"facial|hemifacial|cranial microsomia|asymmetry|dysostosis|anomaly|deformity"|"treacher collins"|goldenhar "tooth|dental malformation|abnormalilties|anomalies|development|formation"|hypodontia|oligodontia|adontia|anodontia|odontogenenis|ameloge
